# Geographic Dialysis Facility Density and Early Dialysis Initiation

**DOI:** 10.1001/jamanetworkopen.2023.50009

**Published:** 2024-01-03

**Authors:** Vagish Hemmige, Priya Deshpande, Keith C. Norris, Jenny I. Shen, Kevin F. Erickson, Kirsten L. Johansen, Ladan Golestaneh

**Affiliations:** 1 Division of Infectious Diseases, Montefiore Medical Center/Albert Einstein College of Medicine, Bronx, New York; 2 Division of Nephrology, Mt Sinai School of Medicine, New York, New York; 3 Department of Medicine, David Geffen School of Medicine, Los Angeles, California; 4 Division of Nephrology, Los Angeles County Harbor-UCLA Medical Center, Los Angeles, California; 5 Division of Nephrology, Baylor College of Medicine, Houston, Texas; 6 Hannepin County Medical Center, Minneapolis, Minnesota; 7 Division of Nephrology, Montefiore Medical Center/Albert Einstein College of Medicine, Bronx, New York

## Abstract

**Importance:**

The decision of when to start maintenance hemodialysis may be affected by health system–level support for high-intensity care as manifested by area dialysis facility density. Yet an association between early hemodialysis initiation and higher area density of dialysis facilities has not been shown.

**Objective:**

To examine whether there is an association between area dialysis facility density and earlier dialysis initiation.

**Design, Setting, and Participants:**

Cross-sectional analysis was conducted of publicly reported claims and geographic-based population data collected in the Medical Evidence files of the US Renal Data System (USRDS), a comprehensive registry of all patients initiating hemodialysis in the US, from calendar years 2011 through 2019. Data were linked to the American Community Survey, using residential zip codes, and then to health service area (HSA) primary care and hospitalization benchmarks, using the Dartmouth Atlas crosswalk. Data were analyzed from November 1, 2021, to August 31, 2023.

**Exposure:**

Dialysis facility density at the level of HSA (number of dialysis facilities per 100 000 HSA residents) split into 5 categories.

**Main Outcomes and Measures:**

The odds of hemodialysis initiation at an estimated glomerular filtration rate (eGFR) greater than 10 mL/min/1.73 m^2^ vs less than or equal to 10 mL/min/1.73 m^2^.

**Results:**

Hemodialysis was initiated in a total of 844 466 individuals at 3397 HSAs at a mean (SD) eGFR of 8.9 (3.8) mL/min/1.73 m^2^. Their mean (SD) age was 63.5 (14.7) years, and 484 346 participants (57.4%) were men. In the HSA category with the highest facility density, individuals were younger (63.3 vs 65.2 years in least-dense HSAs), poorer (mean percent of households living in poverty, 10.4% vs 8.4%), and more commonly had a higher percentage of Black individuals (40.6% vs 11.3%). More individuals in the dialysis-dense HSAs than least-dense HSAs had diabetes (60.1% vs 58.5%) and fewer had access to predialysis nephrology care (60.8% vs 64.1%); the rates of heart failure and immobility varied, but not in a consistent pattern, by HSA dialysis density. The mean (SD) facility density was 4.1 (1.89) centers per 100 000 population in the most dialysis-dense HSAs. Compared with patients in HSAs with a mean of 1.0 per 100 000 population, the odds of hemodialysis initiation at eGFR greater than 10 mL/min/1.73 m^2^ were 1.07 (95% CI, 1.03-1.11) for patients in the densest HSAs, and compared with HSAs with 0 facilities, the odds of early hemodialysis initiation were 1.06 (95% CI, 1.02-1.10) for patients in the densest HSAs.

**Conclusions and Relevance:**

In this cross-sectional study of USRDS- and HSA-level data, HSA dialysis density was associated with early hemodialysis initiation.

## Key Points

**Question:**

Is the density of dialysis facilities within a health service area (HSA) associated with when hemodialysis is initiated in patients?

**Findings:**

In this cross-sectional study of the US Renal Data System linked to the Dartmouth Atlas including 844 466 patients at 3397 HSAs, an association between higher estimated glomerular filtration rate (eGFR) at hemodialysis initiation (early initiation) and HSA-level dialysis density was identified in analyses adjusting for potential confounding variables. Black individuals initiated hemodialysis at a significantly lower eGFR than individuals of other races.

**Meaning:**

The findings of this study suggest there may be an association between higher dialysis facility density and practice patterns that favor early initiation of hemodialysis.

## Teaser

This cross-sectional study examines the time of dialysis initiation in patients with kidney failure living in health service areas with differing dialysis facility density.

## Introduction

Kidney failure prevalence has doubled in the past decade with enormous cost implications for the US health care system.^1,2^ Substantial variability exists across the country on timing of dialysis initiation, as there is no formalized estimated glomerular filtration rate (eGFR) threshold below which hemodialysis is recommended.^3^ Most nephrologists use eGFR as a major factor in decision-making about timing of dialysis initiation,^4^ but as one study observed, decision-making is subject to heuristic thinking^5^ and a dichotomy in management approach, with one approach focused on preventing progression of disease and medical therapy as opposed to the other approach focused on preparation for dialysis. This thinking may also be influenced by surrounding health system practice patterns.^6^

In one study, 11.4% of the total variability attributed to physician decision-making in eGFR at dialysis initiation occurred across physicians, while 88.6% occurred within physicians. The majority of the variability was explained by patient case mix or remained unexplained.^6,7^ Structural aspects of care delivery systems, such as supply and organization of health systems around need, vary widely, especially with regard to the intensity of end-of-life care and prolonged use of dialysis.^7,8^ Medicare’s higher physician reimbursement for dialysis services, compared with chronic kidney disease (CKD) services, may also be a factor favoring dialysis preparation rather than slowing CKD progression.^9,10^ A study of older veterans receiving predialysis care through Medicare vs the Veterans Health Administration (VA)^8^ with the former providing higher reimbursements to physicians for dialysis services than the latter, reported that a higher proportion of patients who received predialysis kidney care in the Medicare system initiated dialysis than patients who received predialysis care in the VA system.^9^ Moreover, health systems that receive dialysis reimbursement through Medicare, compared with the VA, initiated dialysis in patients at significantly higher levels of eGFR.^11^

With this background, the premise of this study is that health service areas (HSAs) that emphasize high-intensity care focused on treating end-organ disease have a higher density of dialysis facilities per capita and may inadvertently initiate hemodialysis earlier than health systems that emphasize preventive care and efforts to mitigate disease progression. We hypothesized that there is an independent association between availability and increased use of dialysis services, with earlier rather than later dialysis initiation indicating overuse.

## Methods

### Study Design

This was a cross-sectional analysis of the US Renal Data System (USRDS)^1^ linked to HSA-level data attributes to test the association of HSA dialysis facility density and odds of early dialysis initiation. We followed the Strengthening the Reporting of Observational Studies in Epidemiology (STROBE) reporting guideline. We obtained institutional review board approval from the Albert Einstein College of Medicine for all analyses. We used data from the USRDS Medical Evidence Report, after obtaining a data use agreement, to examine the eGFR at hemodialysis initiation as reported on the 2728 form among patients with hemodialysis initiated who lived across HSAs in the continental US. Health service areas are self-contained geographic areas defined by the health systems from which they receive care^12^ and those with the highest density of dialysis facilities were compared with HSAs with a lower density of dialysis facilities. Patient residential zip codes were obtained from USRDS and linked to Dialysis Facility Data Archives from Centers for Medicare & Medicaid Services^13^ and to HSA-level ambulatory and hospital characteristics using the zip code/HSA crosswalk available through the Dartmouth Atlas.^14^ These same zip codes within HSAs were linked to the American Community Survey to obtain data on residential race and ethnicity composition and rates of poverty. We linked these data by patient residential zip code to HSA identifier as geographic unit because earlier studies showed health service catchment area characteristics as a better source of information than zip codes with regard to local practice patterns.^14,15^

### Participants

We included incident patients initiating hemodialysis between calendar years 2011 and 2019, using USRDS Medical Evidence forms. We started this cohort in 2011 because of the results of the IDEAL (Initiating Dialysis Early and Late) study^16^ published in 2010 showing equivalent outcomes in patients who started hemodialysis late vs early that marked a shift in nephrologist practice from early to later dialysis initiation. We did not include patients who initiated home dialysis therapies because the timing of home dialysis initiation may vary according to training schedules, trainer availability, and the possibility of exceedingly early initiation in therapies such as incremental peritoneal dialysis. We obtained patient-level sociodemographic and clinical comorbidity variables from the USRDS Medical Evidence files. Race and ethnicity was defined by physicians or administrative staff completing the 2728 forms and likely observed or obtained from the health record. Data on race were provided because race-based health disparities in the US affect patient outcomes and need to be accounted for in efforts to understand trends in health services such as dialysis initiation. Comorbidity data were classified into categories according to logic explained in previous studies using USRDS data.^17^

### Exposure Variable

The exposure variable is the HSA-level density of dialysis facilities, calculated by dividing the number of dialysis facilities in each HSA by the total number of HSA residents (multiplied by 100 000). We categorized the exposure variable into 5 categories. Category 1 had 0 facilities, category 2 represented HSAs with more than 0 and less than the 25th percentile of facility densities, category 3 represented the 25th and less than the 50th percentile of facility densities, category 4 represented the 50th and less than the 75th percentile of facility density, and category 5 represented the highest facility density, or the 75th or greater percentile. We focused on centers and not stations because decision-making by physicians is likely to be influenced by their observation of surrounding center density and because dialysis organizations proactively build or acquire dialysis centers based on establishment of center-based certificates of need.^18^ For the regression models, both continuous and categorized versions of HSA-level dialysis density were used to show the point of inflection for point estimates.

### Outcome Variable

The outcome variable was the eGFR (calculated by CKD-EPI equation 2012)^19^ at dialysis initiation, dichotomized at less than or equal to 10 vs greater than 10 mL/min/1.73 m^2^. We restricted eGFR to less than 20 mL/min/1.73 m^2^ because it is a range targeted by nephrologists for dialysis initiation.^20^ We did not remove the race coefficient from the equations used for this analysis because we were interested in what eGFR data physicians were using to make decisions about dialysis initiation at the time.

### Other Variables

We obtained residential zip code variables on poverty and racial residential make-up and the rural-urban continuum categories from the American Community Survey^20^ and US Department of Agriculture.^21^ We obtained HSA-level data on achievement of primary care benchmarks, including rate of ambulatory-sensitive discharges and rate of diabetic eye examinations, from the Dartmouth Atlas. Per the definition of the Dartmouth Atlas, ambulatory-sensitive discharges are hospitalizations that are preventable when access to primary care is adequate.^12^

### Potential Sources of Bias and Sensitivity Analyses

Because in HSAs with 0 dialysis facilities distance to dialysis and clinical facilities may play a disproportionate role in timing of dialysis initiation, we performed sensitivity analyses in which HSAs with 0 facilities were removed from the exposure variable. We also repeated all regression modeling with the exposure as a continuous variable rather than categorized to validate the primary association. In addition, we repeated analyses using linear regression with the continuous version of eGFR as the outcome variable.

### Missing Data

Albumin and hemoglobin had greater than 5% missing data in the USRDS database. We report on final models with and without albumin and hemoglobin values. Assuming that all missing data were at random, we then used a multiple imputation technique with chained predictive analytics (10 imputations) to report point estimates with imputed and original models^22^ that included albumin and hemoglobin values.

### Statistical Analysis

Data were analyzed from November 1, 2021, to August 31, 2023. We used Stata, version 15.0 (StataCorp LLC) and R, version 4.2.1 (R Project for Statistical Computing) to estimate the proportion of each of patient-level demographic and clinical variables available from USRDS and HSA-level primary care benchmark achievement at each of 5 categories of exposure variable (HSA dialysis facility density). One-way analysis of variance, Kruskal-Wallis, and χ^2^ tests were used to assess the differences in demographic, clinical and population level variables by categories. Analyses were clustered around HSAs. We used mixed linear models to test the variability of eGFR at hemodialysis initiation explained by HSA identifier and, separately, the variability explained by HSA identifier and facility provider in nested models. Because HSA-level dialysis facility density is a nonunique value that can be applied across HSA locations, we were unable to add this variable to the nested models but included it as an adjustment variable.^23^ We then used mixed-effects logit modeling, with random effects intercept assigned to HSA identifier, to examine the association between the odds of eGFR greater than 10 mL/min/1.73 m^2^ at dialysis initiation and HSA density category in unadjusted and progressively adjusted models. In progressive models, adjustments were made for (1) patient age, patient sex, patient race and ethnicity, and year of hemodialysis initiation; (2) type of dialysis access, presence of comorbidities, and etiology of kidney failure; (3) access to predialysis nephrology care, patient insurance, residential area measures of poverty, and proportion of Black residents and rural/urban continuum; and (4) HSA-level success in achieving primary care benchmarks, such as annual rate of hemoglobin A_1c_ measurements and eye examinations among patients with diabetes and rate of ambulatory-sensitive discharges.^24^ Statistical significance was set at a 2-sided α level of .05.

## Results

### Participants and Descriptive Data

**Figure 1.**
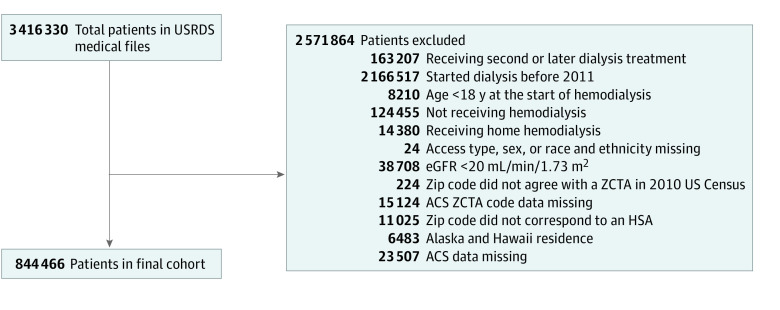
Diagram of Study Cohort ACS indicates American Community Survey; eGFR, estimated glomerular filtration rate; HSA, health service area; USRDS, US Renal Data System; and ZCTA, zip code tabulation area.

**Figure 2.**
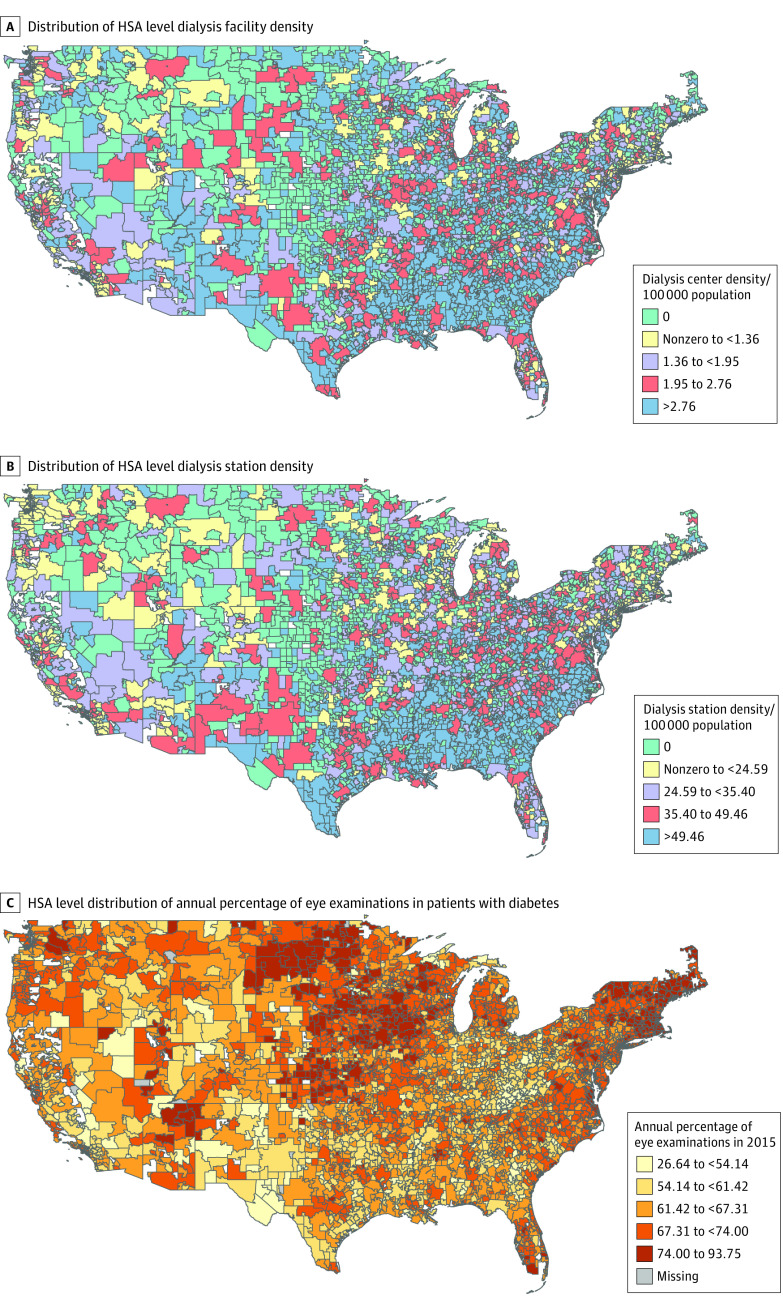
Health Service Area (HSA) Level Findings Health service area–level dialysis facility density (A), HSA-level dialysis station density (B), and HSA-based annual percentage of eye examinations among patients with diabetes in 2015 (C).

There were 3397 HSAs, and the total population of patients receiving incident hemodialysis between 2011 and 2019 meeting our criteria was 844 466 (Figure 1). Women accounted for 42.6% (n = 360 120) and men 57.4% (n = 483 346) of the population; Black individuals represented 28.1%, with Hispanic individuals representing 15.1% of the total. Their mean (SD) age was 63.5 (14.7) years. Overall, 30.0% of individuals had heart failure and 59.1% had diabetes, while 24.1% did not have pre–kidney failure nephrology care. Most individuals (80.5%) initiated hemodialysis with a catheter. The mean (SD) facility density was 4.1 (1.89) centers per 100 000 population in the most dialysis-dense HSAs (Table 1). The median HSA-level dialysis facility density across the country was 1.95 (IQR, 1.36-2.76) centers per 100 000 population, with dialysis-dense HSAs, those with the highest density of dialysis facilities and dialysis stations within facilities, located mostly in areas of the Midwest and Southeast (Figure 2, A and B), and the mean (SD) eGFR at which hemodialysis was initiated between 2011 and 2019 was 8.9 (3.8) mL/min/1.73 m^2^.

**Table 1.** Characteristics of Individuals and HSAs

Characteristic	HSA facility density per 100 000 population					*P* value
	0 (n = 36 947)	>0-1.36 (n = 174 185)	>1.36-1.95 (n = 211 153)	>1.95-2.76 (n = 211 491)	>2.76 (n = 210 690)	
**Patients**						
Age at incidence, mean (SD), y	65.2 (14.1)	64.1 (15.0)	63.2 (14.8)	63.3 (14.7)	63.3 (14.5)	<.001
Sex, No. (%)						
Female	15 265 (41.3)	71 584 (41.1)	88 580 (42.0)	91 114 (43.1)	93 577 (44.4)	<.001
Male	21 682 (58.7)	102 601 (58.9)	122 573 (58.0)	120 377 (56.9)	117 113 (55.6)	
Race, No. (%)						
Asian	661 (1.8)	11 103 (6.4)	10 779 (5.1)	6325 (3.0)	3892 (1.8)	<.001
Black	4182 (11.3)	32 960 (18.9)	49 282 (23.3)	65 073 (30.8)	85 450 (40.6)	
Native American	848 (2.3)	1412 (0.8)	1738 (0.8)	1167 (0.6)	3580 (1.7)	
Pacific Islander	168 (0.4)	2560 (1.5)	2389 (1.1)	1545 (0.7)	988 (0.5)	
White	31 088 (84.1)	126 150 (72.4)	146 965 (69.6)	137 381 (65.0)	116 781 (55.4)	
Ethnicity, No. (%)						
Hispanic	2854 (7.7)	28 286 (16.2)	41 916 (19.8)	37 050 (17.5)	17 204 (8.2)	<.001
Non-Hispanic	34 093 (92.3)	145 899 (83.8)	169 237 (80.2)	174 441 (82.5)	193 486 (91.8)	
Comorbidities, No. (%)						
Heart failure	12 241 (33.1)	52 807 (30.3)	59 297 (28.1)	62 476 (29.5)	66 384 (31.5)	<.001
Coronary disease	7248 (19.6)	31 059 (17.8)	30 201 (14.3)	30 036 (14.2)	28 510 (13.5)	<.001
Diabetes	21 598 (58.5)	99 845 (57.3)	124 362 (58.9)	126 997 (60.0)	126 644 (60.1)	<.001
Cerebrovascular disease	3407 (9.2)	15 054 (8.6)	17 290 (8.2)	19 266 (9.1)	20 635 (9.8)	<.001
Cancer	3417 (9.2)	13 990 (8.0)	14 843 (7.0)	15 090 (7.1)	14 868 (7.1)	<.001
Immobility	6153 (16.7)	25 954 (14.9)	31 974 (15.1)	32 835 (15.5)	33 824 (16.0)	<.001
Alcohol or drug use	914 (2.5)	4497 (2.6)	5871 (2.8)	6003 (2.8)	6042 (2.9)	<.001
Access to pre–kidney failure nephrology care, No. (%)						
No nephrology care	9110 (24.7)	40 631 (23.3)	51 112 (24.2)	52 816 (25.0)	50 227 (23.8)	<.001
Some nephrology care	23 693 (64.1)	110 290 (63.3)	127 382 (60.3)	128 073 (60.6)	128 069 (60.8)	
Unknown	4144 (11.2)	23 264 (13.4)	32 659 (15.5)	30 602 (14.5)	32 394 (15.4)	
Serum albumin, mean (SD), g/dL (n = 562 095)	3.15 (0.70)	3.18 (0.70)	3.16 (0.70)	3.12 (0.70)	3.13 (0.69)	<.001
Serum Hb, mean (SD), g/dL (n = 661 422)	9.5 (1.6)	9.4 (1.7)	9.4 (1.7)	9.3 (1.7)	9.3 (1.7)	<.001
Dialysis access, No. (%)						
Tunneled catheter	29 598 (80.1)	135 574 (94.3)	167 949 (79.5)	170 985 (80.8)	170 737 (81.0)	<.001
Arteriovenous fistula	6445 (17.4)	33 267 (19.1)	36 498 (17.3)	33 756 (16.0)	32 584 (15.5)	
Arteriovenous graft	856 (2.3)	5054 (2.9)	6335 (3.0)	6407 (3.0)	6975 (3.3)	
Other	48 (0.1)	290 (0.2)	371 (0.2)	343 (0.2)	394 (0.2)	
Insurance, No. (%)						
No insurance	1586 (4.3)	8109 (4.7)	12 706 (6.0)	14 995 (7.1)	13 199 (6.3)	<.001
Medicaid	9053 (24.5)	51 812 (29.8)	62 719 (29.7)	56 677 (26.8)	57 092 (27.1)	
Medicare or managed Medicare	20 469 (55.4)	82 438 (47.3)	97 964 (46.4)	103 391 (48.9)	106 551 (50.6)	
Commercial	3747 (10.1)	20 378 (21.9)	23 678 (11.2)	23 358 (11.0)	22 047 (10.5)	
Other (including VA)	2092 (5.7)	11 448 (6.6)	14 086 (6.7)	13 070 (6.2)	11 801 (5.6)	
Presumed etiology of kidney failure, No. (%)						
Diabetes	17 442 (47.2)	83 293 (47.8)	103 592 (49.1)	102 872 (48.6)	98 311 (46.7)	<.001
Glomerulonephritis	2357 (6.4)	12 099 (7.0)	12 436 (5.9)	11 180 (5.3)	10 368 (4.9)	
Hereditary/polycystic kidney disease	895 (2.4)	4060 (2.3)	4216 (2.0)	3783 (1.8)	3331 (1.6)	
Hypertension and cardiac disease	9288 (25.1)	45 575 (26.2)	59 909 (28.4)	64 310 (30.4)	69 892 (33.2)	
Other	6729 (18.2)	28 120 (16.1)	29 885 (14.2)	28 288 (13.4)	27 692 (13.1)	
Transplant failure or complications (solid organ and bone marrow)	236 (0.6)	1038 (0.6)	1115 (0.5)	1058 (0.5)	1096 (0.5)	
**HSA level**						
Socioeconomic attributes						
Zip code level % of Black residents, mean (SD)	6.7 (12.9)	11.4 (17.0)	14.7 (18.7)	21.3 (25.1)	29.6 (29.5)	<.001
Zip code level % of households living below poverty line, mean (SD)	15.5 (8.4)	14.7 (8.9)	17.5 (10.0)	18.6 (10.4)	19.6 (10.4)	<.001
Health care quality metrics						
Rate of discharges for ambulatory-sensitive conditions per 1000 enrollees, mean (SD)	63.7 (28.2)	44.0 (11.4)	46.9 (11.7)	52.2 (13.1)	57.0 (18.5)	<.001
Percentage of women with mammograms within 2 y prior, mean (SD) (n = 844 058)	60.5 (8.8)	61.8 (6.7)	61.2 (6.8)	61.9 (6.3)	61.4 (6.6)	.07
Percentage of enrollees with diabetes with an HbA_1c_ level checked in 2015, mean (SD) (n = 844 266)	85.0 (6.9)	85.7 (3.9)	84.5 (4.2)	85.0 (3.6)	84.3 (6.6)	.002
Annual percentage of eye examinations in 2015 among diabetic enrollees	65.2 (8.4)	68.0 (5.4)	66.0 (5.2)	65.1 (5.2)	64.2 (5.8)	<.001
HSA level total of for-profit dialysis facilities, mean (SD)	0	4.1 (4.9)	10.0 (10.8)	15.0 (22.8)	12.3 (21.1)	<.001
Rural-urban type, No. (%)						
Metropolitan	9186 (39.5)	165 422 (95.0)	193 300 (91.5)	183 870 (86.9)	147 948 (70.2)	<.001
Micropolitan	4978 (13.5)	5433 (3.1)	12 334 (5.8)	21 187 (10.0)	28 576 (16.0)	
Small town	10 485 (28.4)	1636 (0.9)	3082 (1.5)	3530 (1.7)	20 856 (9.9)	
Rural	6898 (18.6)	1694 (1.0)	2437 (1.2)	2904 (1.4)	8298 (3.9)	

Abbreviations: Hb, hemoglobin; HbA_1c_, hemoglobin A_1c_; HSA, health service area; VA, Veterans Administration.

SI conversion factors: To convert albumin to grams per liter, multiply by 10; Hb to grams per liter, multiply by 10; and HbA_1c_ to proportion of total hemoglobin, multiply by 0.01.

### Outcome Data

Individuals with hemodialysis initiated in the most dialysis-dense HSA category were younger (63.3 vs 65.2 years in categories 5 vs 1 of HSA-level dialysis facility density), more commonly women (44.4% vs 41.3%), more commonly Black (40.6% vs 11.3%), and had a higher proportion with diabetes (60.1% vs 58.5%), alcohol or drug use disorder (2.9% vs 2.5%), and cerebrovascular disease (9.8% vs 9.2%) than those in the least dialysis-dense HSAs. Health service areas with the highest dialysis center density had a significantly higher Black residential composition compared with the least-dense HSAs (29.5% vs 12.9%; *P* < .001) and had a higher proportion living below the national poverty line (10.4% in category 5 vs 8.4% in category 1). There was not a consistent pattern of rurality with respect to degree of HSA center density; however, HSAs with 0 dialysis facilities were more commonly designated as small town or rural. Individuals with dialysis initiated in dialysis-dense HSAs had lower rates of engagement with nephrologists before kidney failure (60.8% with some nephrology care in category 5 vs 64.1% in HSAs with 0 dialysis facilities or category 1), while types of insurance varied depending on HSA-level dialysis density but not in a consistent pattern, as did the proportion of patients with heart failure on dialysis initiation with the least-dense HSAs having the highest proportion of patients with heart failure (Table 1). The eGFR at dialysis initiation was higher in dialysis-dense HSAs (eTable 2, eFigure 1, and eFigure 2 in Supplement 1). Across all HSAs, hemodialysis was initiated at lower eGFRs in Black individuals than White individuals (1.20 [95% CI, 1.22-1.18] mL/min/1.73 m^2^ lower in unadjusted models and 1.10 [95% CI, 1.13-1.08] mL/min/1.73 m^2^ lower in fully adjusted models).

There was a higher rate of discharge for ambulatory-sensitive conditions in the HSAs with the highest dialysis density and HSAs with no dialysis facilities (mean [SD], 57.0% [18.5%] for category 5 and 63.7% [28.2%] for category 1 vs 44.0% [11.4%] in category 2). Fewer individuals in HSAs with the highest dialysis density met metrics for ambulatory care quality, such as eye examinations completed for enrollees with diabetes, compared with less dialysis-dense HSAs (Table 1).

### Primary Analysis

In multivariable models with eGFR as a continuous variable, 10% of the total variability of mean eGFR at dialysis initiation was explained by the HSA-level characteristics. There was an association, with odds increasing by progressively higher-density categories, between HSA-level dialysis facility density and odds of hemodialysis initiation at eGFR greater than 10 mL/min/1.73 m^2^ (early). There was a 1.06 (95% CI, 1.02-1.11; *P* =.004) higher odds of initiating hemodialysis in the highest-density HSAs compared with HSAs with 0 dialysis facilities and a 1.07 (95% CI, 1.06-1.07; *P*<.001) higher odds of initiating hemodialysis in the highest-density HSAs compared with category 2 of HSA-based dialysis facility density where the mean density was 1.0 facilities per 100 000 residents (Table 2). The odds remained significantly higher with the addition of comorbidities, albumin levels, and hemoglobin levels, and were not attenuated with the addition of HSA-level measures of health delivery quality and socioeconomic attributes, location with respect to the urban/rural continuum, or patient insurance or access to pre–kidney failure nephrologist care (Table 2).

**Table 2.** Odds of Starting Dialysis Respective to the Density of Dialysis Units in Patients’ Residential HSAs

Variable	Hemodialysis initiated at eGFR >10 mL/min/1.73 m^2^, OR (95% CI)					
	Model 1^a^	*P* value	Model 2^b^	*P* value	Model 3^c^	*P* value
**Regressions included all variables and categories of HSA dialysis facility density**						
Category of HSA dialysis facility density (No. of facilities per 100 000 population)			n = 533 375		n = 533 375	
1 (0)	1 [Reference]		1 [Reference]		1 [Reference]	
2 (>0-1.36)	0.99 (0.95-1.03)	.58	0.99 (0.93-1.03)	.55	1.00 (0.95-1.05)	.94
3 (>1.36-1.95)	1.02 (0.98-1.06)	.29	1.03 (0.99-1.08)	.14	1.04 (1.00-1.05)	.06
4 (>1.95-2.76)	1.03 (1.00-1.07)	.08	1.03 (0.98-1.07)	.21	1.04 (0.99-1.09)	.09
5 (>2.76)	1.06 (1.02-1.10)	.004	1.05 (1.01-1.10)	.003	1.06 (1.02-1.11)	.004
**Regressions without albumin and Hb in models 2 and 3**						
Category of HSA dialysis facility density (No. of facilities per 100 000 population)			n = 844 466		n = 844 4656	
1 (0)	1 [Reference]		1 [Reference]		1 [Reference]	
2 (>0-1.36)	0.99 (0.95-1.03)	.58	1.00 (0.96-1.04)	.91	1.02 (0.97-1.06)	.48
3 (>1.36-1.95)	1.02 (0.98-1.06)	.29	1.03 (0.99-1.07)	.12	1.04 (1.00-1.08)	.04
4 (>1.95-2.76)	1.03 (1.00-1.07)	.08	1.04 (1.00-1.08)	.03	1.05 (1.01-1.10)	.008
5 (>2.76)	1.06 (1.02-1.10)	.004	1.06 (1.02-1.10)	.005	1.07 (1.03-1.11)	.001
**Regressions without category 1 of HSA dialysis facility density (n = 807 519)**						
Category of HSA dialysis facility density (No. of facilities per 100 000 population)						
2 (>0-1.36)	1 [Reference]		1 [Reference]		1 [Reference]	
3 (>1.36-1.95)	1.03 (1.01-1.06)	.007	1.03 (1.00-1.05)	.02	1.05 (1.02-1.08)	.001
4 (>1.95-2.76)	1.05 (1.02-1.08)	.001	1.04 (1.01-1.07)	.004	1.04 (1.01-1.08)	.01
5 (>2.76)	1.07 (1.04-1.10)	<.001	1.06 (1.02-1.09)	<.001	1.07 (1.03-1.11)	<.001

Abbreviations: eGFR, estimated glomerular filtration rate; Hb, hemoglobin; HSA, health service area; OR, odds ratio.

^a^ Multivariate model adjusted for patient demographic factors (age, sex, race and ethnicity, and year of data collection).

^b^ Model 1 variables as well as clinical comorbidities (heart failure, coronary disease, diabetes, cerebrovascular disease, alcohol or drug use, immobility, and institutional residence), access type, serum albumin and serum hemoglobin levels, and cause for dialysis.

^c^ Model 2 variables as well as geographic attributes (patient insurance, access to pre–kidney failure nephrology care, rural-urban continuum, poverty, residential racial makeup, and HSA quality benchmarks).

In a sensitivity analysis in which HSAs with 0 dialysis facilities were excluded from the regression models, the association of HSA-level dialysis density category and odds of dialysis initiation at eGFR greater than 10 mL/min/1.73 m^2^ was significant, with 1.07 (95% CI, 1.03-1.11; *P* < .001) higher odds in HSAs with the highest dialysis facility density compared with HSAs with the lower (category 2) dialysis facility density (Table 2).

### Subgroup and Other Analyses

Because Black individuals were observed to initiate hemodialysis at a significantly lower eGFR than other races across all HSA types in our analyses, we stratified analyses by individual race. In subgroup analyses, we found that the association of HSA-level dialysis facility density and odds of dialysis initiation at an eGFR greater than 10 mL/min/1.73 m^2^ was significant in White individuals, with higher odds of initiating at eGFR greater than 10 mL/min/1.73 m^2^: 1.08 (95% CI, 1.04-1.12; *P* < .001) in unadjusted models and 1.05 (95% CI, 1.00-1.10; *P* = .03) in adjusted models in the highest-density HSAs compared with HSAs with 0 dialysis facilities. In Black individuals, there was also a higher odds of dialysis initiation at eGFR greater than 10 mL/min/1.73 m^2^ (odds ratio, 1.03 [95% CI, 0.94-1.13]; *P* = .50), but this finding was not statistically significant (eTable 1 in Supplement 1). Sensitivity testing that used the continuous version of the exposure variable showed consistent results for the overall and subgroup analyses, and sensitivity analyses that used the continuous version of the outcome variable with the exposure variable as categorical showed a higher eGFR at dialysis initiation in dialysis-dense HSAs compared with less-dense HSAs (eTable 2 and eTable 3 in Supplement 1). Sensitivity analyses that used multiple imputation techniques to account for missing data values for albumin and hemoglobin variables also showed point estimates similar to models in which missing values were present (Table 2).

## Discussion

### Key Results

Our study observed that, in HSAs with the highest dialysis facility density, hemodialysis was initiated earlier than in areas with lower dialysis facility density. Higher dialysis facility density was also associated with higher odds of initiating dialysis at an eGFR greater than 10 mL/min/1.73 m^2^ after eliminating HSAs with no dialysis facilities, where distance to health clinics may have played a role in timing of dialysis initiation, and the odds remained significantly higher after adjustment for patient age, race and ethnicity, sex, comorbidity, etiology of kidney failure, duration of pre–kidney failure nephrology care, vascular access type, and insurance type. The findings were also significant after adjustment for HSA-level benchmarks for quality of health provision, arguing for explanatory factors of early dialysis initiation in dialysis-dense areas not captured by our reported metrics. An example of such an explanatory variable is health system practice patterns, with a pattern of health care systems without strong preventive health care services leading to less medical management of late-stage CKD and thus earlier dialysis initiation.^25,26,27,28^ The HSA-level benchmarks for quality health included in our analyses may not have been strong markers of such a pattern.^8,29^

High-density HSAs across the US were poorer and had a higher proportion of Black residents, but dialysis was initiated in Black individuals later than in individuals from all other racial and ethnic groups. This finding complicates the primary analysis of this study because what is causing delayed dialysis initiation among Black patients may also cause a delay in dialysis initiation in dialysis-dense HSAs serving a predominantly Black patient population. In our subgroup analysis, however, early initiation of hemodialysis in dialysis-dense HSAs applied to both Black and White individuals. To our knowledge, the delay in dialysis initiation among Black patients with kidney failure is a novel finding.

### Interpretations and Generalizability

Health service area–level variability in dialysis initiation practice patterns has been previously described, and a 2014 study used this variability as an instrumental variable to estimate risk of mortality with timing of dialysis initiation across the US.^30^ This study found that in areas with greater market competition, dialysis was initiated earlier than in other areas and that the variation in timing of dialysis initiation was present across subgroups of patients with heart failure, diabetes, and those without insurance, suggesting that patient comorbidity alone does not fully explain this variability. Similarly, in our analysis there was not a consistent pattern wherein dialysis-dense communities had a higher rate of conditions that would encourage early dialysis initiation, such as heart failure, older age, and immobility; furthermore, the addition of the comorbidity variables to our regression models did not attenuate the primary association. However, addition of HSA quality metrics to the model did not attenuate the association either, making the case for uncaptured reasons for local practice patterns as explanatory factors.

Variations in appropriateness and quality of care between physicians and health systems provide an opportunity to improve quality and equity across geographic entities.^6,25,31^ With the publication of the IDEAL trial,^16^ a randomized clinical trial that showed that earlier dialysis initiation did not improve patient outcomes, the early dialysis initiation trend that was present for the previous decade reversed after 2011.^7^ The eGFR at which dialysis is initiated became more dependent on physician decision-making after consideration of clinical signs and symptoms, availability of dialysis access, and patient preference.^26,27^ Some nephrology groups entered into financial arrangements with dialysis providers wherein they shared oversight and financial responsibility for dialysis facility census and performance^28^ and for physicians who had a financial stake in performance of area dialysis facilities, earlier initiation of dialysis would be one way to improve financial performance.^3^ Dialysis facility-dense HSAs may be underresourced and more likely to have safety-net health care systems with fragmented ambulatory care that pose disproportionate risks to patients with complicated conditions and illness, such as those with CKD. Physicians working in such underresourced systems may find dialysis facilities provide more structured, comprehensive care for their patients than the safety-net ambulatory care settings that provide care for patients before dialysis is initiated.^8,15^ For this reason, they may start dialysis in patients with multiple conditions who otherwise may be unrecognized earlier than is absolutely necessary by clinical signs and symptoms.^32^ Continuation of unplanned hemodialysis initiated in the setting of hospitalization for patients with advanced CKD and concomitant acute kidney injury may be the easiest approach postdischarge.^33,34^ In these cases, hospitalization may be viewed by physicians as an opportunity to coordinate dialysis initiation or as a requirement in advance of a surgical or angiographic procedure.^25,35^ Physicians operating within inadequate ambulatory services come to rely on the standard structure of care of dialysis facilities and a normalized referral pathway for patients with late-stage CKD and more complex comorbidities.^36,37^ Wong et al^5^ described physician decision-making regarding timing of dialysis initiation as primarily dependent on sources of momentum and dynamic interactions with patients. Physician practice and emphasis on preparing for dialysis as opposed to forestalling kidney disease progression is a factor involved in early dialysis initiation. Because mitigating kidney failure progression will depend on a robust and coordinated ambulatory care system to engage patients early and diagnose illness and initiate and maximally titrate guideline-based medications, improving ambulatory reach in HSAs better equipped with kidney failure treatment vs preventive care services is one strategy to improve outcomes for patients experiencing kidney disease.

### Limitations

Our study has several limitations. The clinical metrics reported on the 2728 form may be inaccurate, and evidence suggests that factors not on the Medical Evidence Report may mediate dialysis initiation time, indicating residual confounding as a factor in earlier dialysis initiation.^7^ Another potential source of bias is the creatinine level entered by individuals completing the 2728 form and whether it is systematically lower in patients with dialysis initiated in the hospital setting after a few dialysis sessions compared with the outpatient setting. Indeed, a large residual variance was left unexplained by HSA- and facility-level characteristics in our study, but as other studies have observed, the eGFR visible to treating physicians and the environment within which they practice may influence their decision-making.^4,5,6,31^ We could not account for patients who did not initiate hemodialysis when indicated. Furthermore, we were unable to ascertain the degree to which distance from dialysis facilities was associated with eGFR at dialysis initiation but observed an association between facility density and early dialysis initiation across rural, micropolitan, and metropolitan areas. In addition, HSAs with the highest dialysis facility density may also have the highest prevalence of patients with kidney failure in need of these facilities and where fewer patients had adequate dialysis preparation, resulting in a higher incidence of patients who present to the emergency department with symptoms of kidney failure, such as uremia or fluid overload, necessitating dialysis initiation on an emergent basis. It is unclear, however, if this would appear as if dialysis was initiated in those patients earlier than in patients who were prepared for dialysis. Because the proportion of patients with heart failure and immobility, both reasons for which dialysis may be initiated early, was not consistently higher as dialysis density increased, and because the addition of these potential confounders to the multivariable model did not significantly attenuate the primary association, it is unlikely that higher incidence of kidney failure alone explains the association of dialysis facility density and early dialysis initiation.

In our analysis, dialysis-dense HSAs were poorer and less frequently met ambulatory care benchmarks than other HSAs. Health service areas with 0 facilities, like dialysis-dense HSAs, had higher rates of ambulatory hospitalizations and heart failure than other HSA types. Because of their locations in rural communities or small towns, 0 facility HSAs likely face barriers, such as long distance to quality ambulatory care that may have been responsible for these results and that introduced complexity in the comparisons of the different HSAs. Yet despite this similarity, high-density HSAs initiated hemodialysis in patients at higher eGFRs than HSAs with 0 facilities, supporting an independent role for area dialysis density as a factor considered in early initiation. In addition, in sensitivity analyses in which 0 facility HSAs were eliminated, the association between density and early dialysis initiation remained. Health service area as a geographic allocation for dialysis facility density may not accurately identify local practice patterns; however, timing of dialysis initiation reflects clinical behaviors of a hospital catchment’s workforce as shown by the Dartmouth Atlas.^12^

## Conclusions

In this cross-sectional study, HSA-based higher density of dialysis facilities was associated with earlier dialysis initiation among an incident kidney failure population. This finding lends support to the notion that dialysis facility saturation of HSAs may represent practice patterns that affect timing of hemodialysis initiation.

## Notes

Supplement 1. **eTable 1.** Regression Modeling Stratified by Individual Race **eTable 2.** Regression Modeling for Total Population and Stratified by Individual Race With Exposure as Continuous Variable **eTable 3.** Linear Regression Modeling Where Outcome Variable Is Continuous **eFigure 1.** Scatter Plot of eGFR at Dialysis Initiation at the HSA Level Dialysis Facility Density in 1% Random Sample of the Dataset **eFigure 2.** Scatter Plot of eGFR at Dialysis Initiation in Those Diagnosed With Heart Failure at the HSA Level Dialysis Facility Density in 1% Random Sample of the Dataset

Supplement 2.
Data Sharing Statement

## References

[1] United States Renal Data System. 2022 USRDS Annual Data Report: Epidemiology of Kidney Disease in the United States. National Institutes of Health, National Institute of Diabetes and Digestive and Kidney Diseases; 2022.

[2] Medicare Program; Revisions to Payment Policies Under the Physician Fee Schedule and Other Revisions to Part B for CY 2018; Medicare Shared Savings Program Requirements; and Medicare Diabetes Prevention Program. Office of the Federal Register, National Archives and Records Administration; 2017. 29231695

[3] Berns JS, Saffer TL, Lin E. Addressing financial disincentives to improve CKD care. J Am Soc Nephrol. 2018;29(11):2610-2612. doi:10.1681/ASN.2018040438 30305309 PMC6218867

[4] van de Luijtgaarden MW, Noordzij M, Tomson C, . Factors influencing the decision to start renal replacement therapy: results of a survey among European nephrologists. Am J Kidney Dis. 2012;60(6):940-948. doi:10.1053/j.ajkd.2012.07.015 22921638

[5] Wong SP, Vig EK, Taylor JS, . Timing of initiation of maintenance dialysis: a qualitative analysis of the electronic medical records of a national cohort of patients from the Department of Veterans Affairs. JAMA Intern Med. 2016;176(2):228-235. doi:10.1001/jamainternmed.2015.7412 26809745 PMC4758379

[6] Sood MM, Manns B, Dart A, ; Canadian Kidney Knowledge Translation and Generation Network (CANN-NET). Variation in the level of eGFR at dialysis initiation across dialysis facilities and geographic regions. Clin J Am Soc Nephrol. 2014;9(10):1747-1756. doi:10.2215/CJN.12321213 25248743 PMC4186523

[7] Li Y, Jin Y, Kapke A, . Explaining trends and variation in timing of dialysis initiation in the United States. Medicine (Baltimore). 2017;96(20):e6911. doi:10.1097/MD.0000000000006911 28514305 PMC5440142

[8] O’Hare AM, Rodriguez RA, Hailpern SM, Larson EB, Kurella Tamura M. Regional variation in health care intensity and treatment practices for end-stage renal disease in older adults. JAMA. 2010;304(2):180-186. doi:10.1001/jama.2010.924 20628131 PMC3477643

[9] Kahrass H, Strech D, Mertz M. The full spectrum of clinical ethical issues in kidney failure: findings of a systematic qualitative review. PLoS One. 2016;11(3):e0149357. doi:10.1371/journal.pone.0149357 26938863 PMC4777282

[10] Bennett WM. Ethical conflicts for physicians treating ESRD patients. Semin Dial. 2004;17(1):1-3. doi:10.1111/j.1525-139X.2004.17102.x 14717800

[11] Yu MK, O’Hare AM, Batten A, . Trends in timing of dialysis initiation within versus outside the Department of Veterans Affairs. Clin J Am Soc Nephrol. 2015;10(8):1418-1427. doi:10.2215/CJN.12731214 26206891 PMC4527039

[12] Dartmouth Atlas Project. Glossary. 2023. Accessed October 4, 2023. https://www.dartmouthatlas.org/glossary/

[13] CMS.gov. Dialysis facilities data archive. 2016-2022. Accessed October 25, 2023. https://data.cms.gov/provider-data/topics/dialysis-facilities/data-sources

[14] Wennberg JE, Cooper MM. The Dartmouth Atlas of Health Care in the New England States: The Center for the Evaluative Clinical Sciences. American Hospital Publishing, Inc; 1996. 36758128

[15] Nguyen C, Chernew M, Ostrer I, Beaulieu N. Comparison of healthcare delivery systems in low- and high-income communities. December 23, 2019. Accessed November 22, 2023. https://www.ajmc.com/view/comparison-of-healthcare-delivery-systems-in-low-and-highincome-communities

[16] Cooper BA, Branley P, Bulfone L, ; IDEAL Study. A randomized, controlled trial of early versus late initiation of dialysis. N Engl J Med. 2010;363(7):609-619. doi:10.1056/NEJMoa1000552 20581422

[17] Erickson KF, Zhao B, Niu J, . Association of hospitalization and mortality among patients initiating dialysis with hemodialysis facility ownership and acquisitions. JAMA Netw Open. 2019;2(5):e193987. doi:10.1001/jamanetworkopen.2019.3987 31099872 PMC6537810

[18] Levin DI, Lingham T, Janiga NJ. 2020 Outlook: dialysis clinics and ESRD. FM Vantage Point. March 6, 2020. Accessed November 22, 2023. https://healthcareappraisers.com/2020-outlook-dialysis-clinics-and-esrd/

[19] Inker LA, Schmid CH, Tighiouart H, ; CKD-EPI Investigators. Estimating glomerular filtration rate from serum creatinine and cystatin C. N Engl J Med. 2012;367(1):20-29. doi:10.1056/NEJMoa1114248 22762315 PMC4398023

[20] US Census Bureau. American Community Survey data. 2022. Accessed October 25, 2023. https://www.census.gov/programs-surveys/acs/data.html

[21] US Department of Agriculture. Economic Research Service. Rural-urban continuum codes. Accessed November 27, 2023. https://www.ers.usda.gov/data-products/rural-urban-continuum-codes/

[22] Jakobsen JC, Gluud C, Wetterslev J, Winkel P. When and how should multiple imputation be used for handling missing data in randomised clinical trials—a practical guide with flowcharts. BMC Med Res Methodol. 2017;17(1):162. doi:10.1186/s12874-017-0442-1 29207961 PMC5717805

[23] Schunck R, Perales F. Within- and between-cluster effects in generalized linear mixed models: a discussion of approaches and the Xthybrid command. Stata J. 2017;17(1):89-115. doi:10.1177/1536867X1701700106

[24] Browne WJ, Subramanian SV, Jones K, Goldstein H. Variance partitioning in multilevel logistic models that exhibit overdispersion. J R Stat Soc Ser A Stat Soc. 2005;168(3):599-613. doi:10.1111/j.1467-985X.2004.00365.x

[25] Streja E, Nicholas SB, Norris KC. Controversies in timing of dialysis initiation and the role of race and demographics. Semin Dial. 2013;26(6):658-666. doi:10.1111/sdi.12130 24102770 PMC3836868

[26] Price LC, Arnold C. Kidney dialysis is a booming business—is it also a rigged one? Sci Am. December 14, 2020. Accessed November 22, 2023. https://www.scientificamerican.com/article/kidney-dialysis-is-a-booming-business-is-it-also-a-rigged-one1/

[27] Muscat DM, Kanagaratnam R, Shepherd HL, Sud K, McCaffery K, Webster A. Beyond dialysis decisions: a qualitative exploration of decision-making among culturally and linguistically diverse adults with chronic kidney disease on haemodialysis. BMC Nephrol. 2018;19(1):339. doi:10.1186/s12882-018-1131-y 30482170 PMC6258454

[28] Glickman A, Lin E, Berns JS. Conflicts of interest in dialysis: a barrier to policy reforms. Semin Dial. 2020;33(1):83-89. doi:10.1111/sdi.12848 31899827 PMC7237383

[29] O’Hare AM, Wong SP, Yu MK, . Trends in the timing and clinical context of maintenance dialysis initiation. J Am Soc Nephrol. 2015;26(8):1975-1981. doi:10.1681/ASN.2013050531 25700539 PMC4520153

[30] Scialla JJ, Liu J, Crews DC, ; DEcIDE Network Patient Outcomes in End Stage Renal Disease Study Investigators. An instrumental variable approach finds no associated harm or benefit with early dialysis initiation in the United States. Kidney Int. 2014;86(4):798-809. doi:10.1038/ki.2014.110 24786707 PMC4182128

[31] Sood MM, Komenda P, Rigatto C, Hiebert B, Tangri N. The association of eGFR reporting with the timing of dialysis initiation. J Am Soc Nephrol. 2014;25(9):2097-2104. doi:10.1681/ASN.2013090953 24652801 PMC4147980

[32] Crews DC, Scialla JJ, Liu J, ; Developing Evidence to Inform Decisions About Effectiveness (DEcIDE) Patient Outcomes in End Stage Renal Disease Study Investigators. Predialysis health, dialysis timing, and outcomes among older United States adults. J Am Soc Nephrol. 2014;25(2):370-379. doi:10.1681/ASN.2013050567 24158988 PMC3904572

[33] Laham G, Pujol GS, Guzman J, Boccia N, Abib A, Diaz CH. Early start hemodialysis with a catheter may be associated with greater mortality: a propensity score analysis. Semin Dial. 2023;36(4):294-302. doi:10.1111/sdi.13157 37088891

[34] Chan CT, Blankestijn PJ, Dember LM, ; Conference Participants. Dialysis initiation, modality choice, access, and prescription: conclusions from a Kidney Disease: Improving Global Outcomes (KDIGO) controversies conference. Kidney Int. 2019;96(1):37-47. doi:10.1016/j.kint.2019.01.017 30987837

[35] Hao H, Lovasik BP, Pastan SO, Chang HH, Chowdhury R, Patzer RE. Geographic variation and neighborhood factors are associated with low rates of pre-end-stage renal disease nephrology care. Kidney Int. 2015;88(3):614-621. doi:10.1038/ki.2015.118 25901471

[36] Song Z, Kannan S, Gambrel RJ, . Physician practice pattern variations in common clinical scenarios within 5 US metropolitan areas. JAMA Health Forum. 2022;3(1):e214698. doi:10.1001/jamahealthforum.2021.4698 35977237 PMC8903123

[37] Slinin Y, Guo H, Li S, . Provider and care characteristics associated with timing of dialysis initiation. Clin J Am Soc Nephrol. 2014;9(2):310-317. doi:10.2215/CJN.04190413 24436477 PMC3913233

