# Supplementary material for: Geographic Dialysis Facility Density and Early Dialysis Initiation
Source: JAMA Netw Open. 2024 Jan 3;7(1):e2350009. doi: 10.1001/jamanetworkopen.2023.50009 (PMC10765261; doi:10.1001/jamanetworkopen.2023.50009)
Supplement: Supplement 1. — eTable 1. Regression Modeling Stratified by Individual Race eTable 2. Regression Modeling for Total Population and Stratified by Individual Race With Exposure as Continuous Variable eTable 3. Linear Regression Modeling Where Outcome Variable Is Continuous eFigure 1. Scatter Plot of eGFR at Dialysis Initiation at the HSA Level Dialysis Facility Density in 1% Random Sample of the Dataset eFigure 2. Scatter Plot of eGFR at Dialysis Initiation in Those Diagnosed With Heart Failure at the HSA Level Dialysis Facility Density in 1% Random Sample of the Dataset [file jamanetwopen-e2350009-s001.pdf]

Supplementary Online Content

Hemmige V, Deshpande P, Norris KC, et al. Geographic Dialysis Facility Density and Early Dialysis Initiation. *JAMA Netw Open*. 2024;7(1):e2350009. doi:10.1001/jamanetworkopen.2023.50009

eTable 1. Regression Modeling Stratified by Individual Race

eTable 2. Regression Modeling for Total Population and Stratified by Individual Race With Exposure as Continuous Variable

eTable 3. Linear Regression Modeling Where Outcome Variable Is Continuous

eFigure 1. Scatter Plot of eGFR at Dialysis Initiation at the HSA Level Dialysis Facility Density in 1% Random Sample of the Dataset

eFigure 2. Scatter Plot of eGFR at Dialysis Initiation in Those Diagnosed With Heart Failure at the HSA Level Dialysis Facility Density in 1% Random Sample of the Dataset

This supplementary material has been provided by the authors to give readers additional information about their work.

© 2024 Hemmige V et al. *JAMA Network Open.*

eTable 1. Regression modeling stratified by individual race

Odd of starting HD at GFR > 10 mL/min/1.73cm^2 (CI)	Model 1: Multivariate model adjusted for patient demographic factors (age, sex and race/ethnicity and year of data collection)	P value	Model 2: Model 1+ clinical comorbidity, , access type, serum albumin and serum Hgb and dialysis cause	P value	Model 3: Model 2 + geographic attributes (Patients insurance, access to pre-ESRD nephrology care, rural-urban continuum, poverty, residential racial makeup, HSA quality benchmarks)	P value
Regression of full model with Albumin and Hemoglobin, Black individuals only (n=146,386)						
Category of HSA facility density	N=236,947					
1 (0 facilities)	1		1		1	
2(>0-1.36)	1.03(0.94-1.14)	0.5	1.12(0.99-1.26)	0.07	1.04(0.91-1.18)	0.6
3(1.36-1.95)	1.04(0.95-1.15)	0.4	1.12(1.0-1.26)	0.06	1.06(0.93-1.19)	0.4
4(1.05-2.76)	1.06(0.97-1.17)	0.2	1.12(0.99-1.26)	0.06	1.07(0.96-1.20)	0.3
5(>2.76)	1.03(0.94-1.13)	0.5	1.07(0.96-1.20)	0.3	1.05(0.93-1.18)	0.4
Regression of full model without Albumin and Hemoglobin, White individuals only (n=355,564)						
Category of HSA facility density	N=558,364					
1 (0 facilities)	1		1		1	
2(>0-1.36)	0.97(0.93-1.01)	0.2	0.96(0.91-1.01)	0.1	1.01(0.96-1.05)	0.8
3(1.36-1.95)	1.02(0.98-1.06)	0.4	1.02(0.99-1.07)	0.3	1.06(1.01-1.10)	0.02
4(1.05-2.76)	1.03(0.99-1.07)	0.1	1.02(0.98-1.07)	0.4	1.05(1.00-1.10)	0.03
5(>2.76)	1.08(1.04-1.12)	<0.001	1.07(1.02-1.12)	0.004	1.05(1.00-1.10)	0.03

© 2024 Hemmige V et al. *JAMA Network Open.*

eTable 2. Regression modeling for total population and stratified by individual race with exposure as continuous variable

Odd of starting HD at GFR > 10 mL/min/1.73cm^2 (CI)	Model 1: Multivariate model adjusted for patient demographic factors (age, sex and race/ethnicity and year of data collection)	P value	Model 2: Model 1+ clinical comorbidity, , access type, serum albumin and serum Hgb and dialysis cause N=533,375	P value	Model 3: Model 2 + geographic attributes (Patients insurance, access to pre-ESRD nephrology care, rural-urban continuum, poverty, residential racial makeup, HSA quality benchmarks) N=533,375	P value
For every 1 unit increase in HSA level dialysis facility density	1.01(1.00-1.02)	0.001	1.01(1.00-1.02)	0.05	1.01(1.00-1.02)	0.004
Regressions without Albumin and Hemoglobin in Models 2 and 3 (n=844,467)						
For every 1 unit increase in HSA level dialysis facility density	1.01(1.00-1.01)	0.001	1.01(1.00-1.01)	0.008	1.02(1.01-1.02)	<0.001
Regressions with the full model and multiple imputation used for variables with missing data (Albumin and Hemoglobin); n=533,375						
For every 1 unit increase in HSA level dialysis facility density	1.01(1.00-1.01)	0.001	1.01(.00-1.01)	0.04	1.01(1.00-1.02)	0.004
Regression of full model without Albumin and Hemoglobin, Black individuals only (n=235,947)						

© 2024 Hemmige V et al. *JAMA Network Open.*

For every 1 unit increase in HSA level dialysis facility density	0.99(0.98-1.00)	0.1	0.99(0.98-1.00)	0.03	1.00(0.99-1.01)	0.8
Regression of full model without Albumin and Hemoglobin, White individuals only (n=558,364)						
For every 1 unit increase in HSA level dialysis facility density	1.02(1.01-1.02)	<0.001	1.01(1.01-1.02)	<0.001	1.02(1.01-1.02)	<0.001

© 2024 Hemmige V et al. *JAMA Network Open.*

eTable 3. Linear regression modeling where outcome variable is continuous.

Increase in eGFR at dialysis initiation	Model 1: Multivariate model adjusted for patient demographic factors (age, sex and race/ethnicity and year of data collection)	P value	Model 2: Model 1+ clinical comorbidity, , access type, serum albumin and serum Hgb and dialysis cause	P value	Model 3: Model 2 + geographic attributes (Patients insurance, access to pre-ESRD nephrology care, rural-urban continuum, poverty, residential racial makeup, HSA quality benchmarks)	P value
Regression of full model with albumin and hemoglobin (n=533,053)						
Category of HSA facility density	N=866,467					
1 (0 facilities)	1		1		1	
2(>0-1.36)	-0.02(-0.10-+0.05)	0.5	-0.08(-0.15-+0.006)	0.7	-0.02(-0.1-+0.07)	0.7
3(1.36-1.95)	0.4(-0.02-0.12)	0.2	0.04(-0.04-0.13)	0.3	0.07(-0.01-+0.15)	0.07
4(1.05-2.76)	0.08(0.01-0.15)	0.02	0.04(-0.04-0.13)	0.3	0.07(0.003-+0.15)	0.06
5(>2.76)	0.11(0.04-0.17)	0.002	0.11(0.29-0.19)	0.008	0.11(0.03-0.18)	0.006
Regressions with 0 facility HSAs eliminated ;(n=508,667)						
Category of HSA facility density	N=807,519					
2(>0-1.36)	1		1		1	
3(1.36-1.95)	0.07(0.03-0.11)	0.001	0.09(0.04-0.14)	<0.001	0.09(0.04-0.14)	<0.001
4(1.05-2.76)	0.11(0.06-0.15)	<0.001	0.09(0.04-0.15)	0.001	0.09(0.03-0.14)	0.001
5(>2.76)	0.14(0.08-0.19)	<0.001	0.13(0.06-0.19)	<0.001	0.13(0.06-0.19)	<0.001

© 2024 Hemmige V et al. *JAMA Network Open.*

eFigure 1. Scatter plot of eGFR at dialysis initiation at the HSA level dialysis facility density in 1% random sample of the dataset

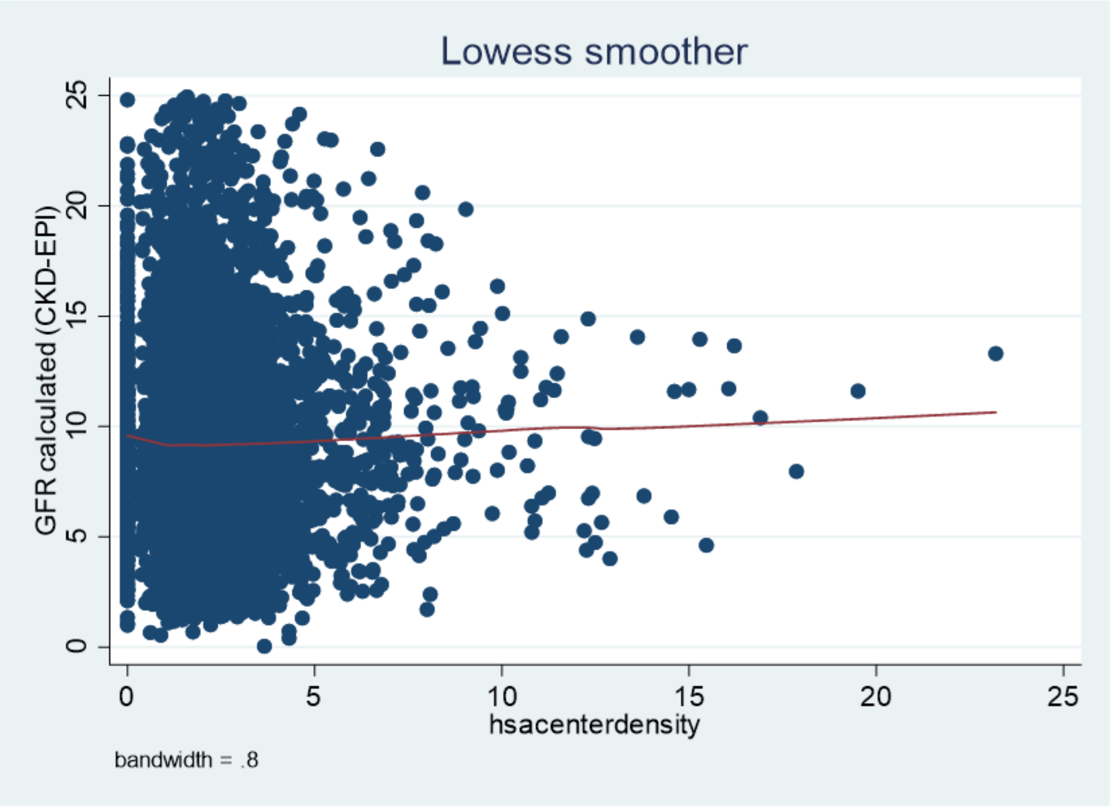

© 2024 Hemmige V et al. *JAMA Network Open.*

eFigure 2. Scatter plot of eGFR at dialysis initiation in those diagnosed with heart failure at the HSA level dialysis facility density in 1% random sample of the dataset

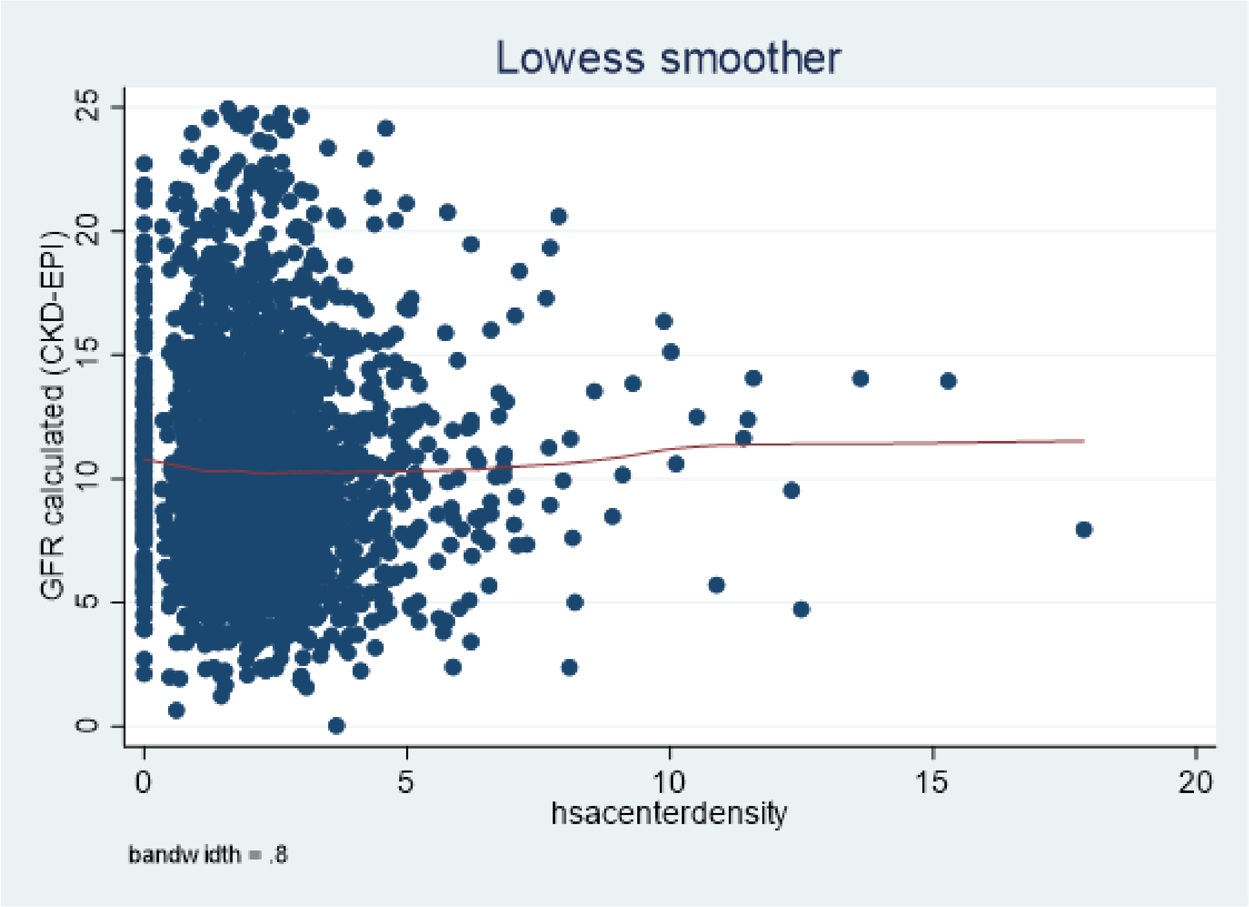

© 2024 Hemmige V et al. *JAMA Network Open.*
