# Supplementary material for: Geographic Dialysis Facility Density and Early Dialysis Initiation
Source: JAMA Netw Open. 2024 Jan 3;7(1):e2350009. doi: 10.1001/jamanetworkopen.2023.50009 (PMC10765261; doi:10.1001/jamanetworkopen.2023.50009)
Supplement: Supplement 2. — Data Sharing Statement [file jamanetwopen-e2350009-s002.pdf]

Data Sharing Statement

Hemmige. Geographic Dialysis Facility Density and Early Dialysis Initiation. *JAMA Netw Open*. Published January 03, 2024. doi:10.1001/jamanetworkopen.2023.50009

Data
Data available: No

Additional Information
Explanation for why data not available: The data is freely available at the USRDS with a data use agreement. I don't think I can share USRDS data without permission from NIDDK.
